# A machine learning-assisted system to predict thyrotoxicosis using patients’ heart rate monitoring data: a retrospective cohort study

**DOI:** 10.1038/s41598-023-48199-x

**Published:** 2023-11-30

**Authors:** Kyubo Shin, Jongchan Kim, Jaemin Park, Tae Jung Oh, Sung Hye Kong, Chang Ho Ahn, Joon Ho Moon, Min Joo Kim, Jae Hoon Moon

**Affiliations:** 1 THYROSCOPE INC., Ulsan, Republic of Korea; 2 Department of Internal Medicine, Seoul National University Bundang Hospital and Seoul National University College of Medicine, Seongnam, Republic of Korea; 3 Center for Artificial Intelligence in Healthcare, Seoul National University Bundang Hospital, Seongnam, Republic of Korea

**Keywords:** Thyroid diseases, Machine learning

## Abstract

Previous studies have shown a correlation between resting heart rate (HR) measured by wearable devices and serum free thyroxine concentration in patients with thyroid dysfunction. We have developed a machine learning (ML)-assisted system that uses HR data collected from wearable devices to predict the occurrence of thyrotoxicosis in patients. HR monitoring data were collected using a wearable device for a period of 4 months in 175 patients with thyroid dysfunction. During this period, 3 or 4 thyroid function tests (TFTs) were performed on each patient at intervals of at least one month. The HR data collected during the 10 days prior to each TFT were paired with the corresponding TFT results, resulting in a total of 662 pairs of data. Our ML-assisted system predicted thyrotoxicosis of a patient at a given time point based on HR data and their HR-TFT data pair at another time point. Our ML-assisted system divided the 662 cases into either thyrotoxicosis and non-thyrotoxicosis and the performance was calculated based on the TFT results. The sensitivity, specificity, positive predictive value (PPV), and negative predictive value (NPV) of our system for predicting thyrotoxicosis were 86.14%, 85.92%, 52.41%, and 97.18%, respectively. When subclinical thyrotoxicosis was excluded from the analysis, the sensitivity, specificity, PPV, and NPV of our system for predicting thyrotoxicosis were 86.14%, 98.28%, 94.57%, and 95.32%, respectively. Our ML-assisted system used the change in mean, relative standard deviation, skewness, and kurtosis of HR while sleeping, and the Jensen–Shannon divergence of sleep HR and TFT distribution as major parameters for predicting thyrotoxicosis. Our ML-assisted system has demonstrated reasonably accurate predictions of thyrotoxicosis in patients with thyroid dysfunction, and the accuracy could be further improved by gathering more data. This predictive system has the potential to monitor the thyroid function status of patients with thyroid dysfunction by collecting heart rate data, and to determine the optimal timing for blood tests and treatment intervention.

## Introduction

Thyrotoxicosis is a clinical syndrome resulting from the high concentration of free thyroxine (T4) and/or free triiodothyronine (T3). The prevalence of thyrotoxicosis is approximately 2%, and its most common cause (60–90%) is Graves’ disease, an autoimmune disease that stimulates the thyroid gland to produce and release thyroid hormone^1–3^. Thyrotoxicosis cannot be diagnosed with clinical symptoms and signs alone because there are various non-specific symptoms and signs such as fatigue, anxiety, palpitations, sweating, sleep disturbance, and weight loss^4^. In real clinical practice, blood tests for serum thyroid hormone levels (thyroid function test, TFT) are required for diagnosing thyrotoxicosis and the treatment goal is to maintain normal TFT results in the patients^5^. Since radioimmunoassay is generally required for TFT, unlike diabetic patients who can measure blood glucose by themselves, patients with thyroid dysfunction do not have an objective indicator to check their disease status.

Thyroid hormone has a positive chronotropic and inotropic effect. It stimulates the rate and force of systolic contraction and the rate of diastolic relaxation^6^. Therefore, thyrotoxicosis increases systolic blood pressure and heart rate (HR) and widens pulse pressure^7^. Although palpitation and increased HR are among the most frequent symptoms and signs of thyrotoxicosis^6^, and the easiest to quantify, HR can easily be affected by other factors such as the patient’s emotional state, body position, and physical activities. To overcome this limitation of HR parameters, our previous study demonstrated that resting HR monitored by a wearable device that can measure the user’s HR and physical activity using photoplethysmography and a 3-axis accelerometer is associated with serum free T4 level in patients with thyrotoxicosis^8^. This means that HR parameters from ‘high definition’ data obtained by wearable devices can be an objective indicator for monitoring the thyroid function status of the patients.

In this study, we expanded our previous study and developed a machine learning (ML)-assisted system to predict thyrotoxicosis using patients’ HR data monitored by wearable devices. Through this study, it will be possible to develop digital therapeutics that can be prescribed in actual clinical practice for monitoring thyroid function status of patients with thyroid dysfunction.

## Results

### Model validation

The performance of our ML-assisted system for detecting thyrotoxicosis was summarized in Tables 2 and 3. Our ML-assisted system divided 662 cases into thyrotoxicosis or non-thyrotoxicosis. The sensitivity, specificity, PPV, and NPV of our system to predict thyrotoxicosis were 86.14%, 85.92%, 52.41%, and 97.18%, respectively (Table 1). In the 79 cases which showed false positive results, 74 cases were subclinical thyrotoxicosis. When subclinical thyrotoxicosis was excluded in the analysis, the sensitivity, specificity, PPV, and NPV of our system to predict thyrotoxicosis were 86.14%, 98.28%, 94.57%, and 95.32%, respectively (Table 2).

**Table 1:** Prediction of thyrotoxicosis by the ML-assisted system.

	Predicted thyroid function state	
	Thyrotoxicosis	Non-thyrotoxicosis
True thyroid function state		
Thyrotoxicosis	87	14
Non-thyrotoxicosis	79	482

The ML-assisted system divided 662 data into thyrotoxicosis or non-thyrotoxicosis (predicted thyroid function state). True thyroid function state is based on the thyroid function test and thyrotoxicosis was defined as serum free T4 level > 1.78 ng/dL. *ML* machine learning.

**Table 2:** Prediction of thyrotoxicosis by the ML-assisted system excluding subclinical thyrotoxicosis.

	Predicted thyroid function state	
	Thyrotoxicosis	Non-thyrotoxicosis
True thyroid function state		
Thyrotoxicosis	87	14
Non-thyrotoxicosis	5	285

Among 662 data, 263 data of subclinical thyrtoxicosis defined as serum free T4 level of 0.89–1.78 ng/dL and serum TSH level < 0.3 mIU/L were excluded in the analysis. The ML-assisted system divided 399 data into thyrotoxicosis or non-thyrotoxicosis (predicted thyroid function state). True thyroid function state is based on the thyroid function test and thyrotoxicosis was defined as serum free T4 level > 1.78 ng/dL. ML, machine learning.

**Figure 1:**
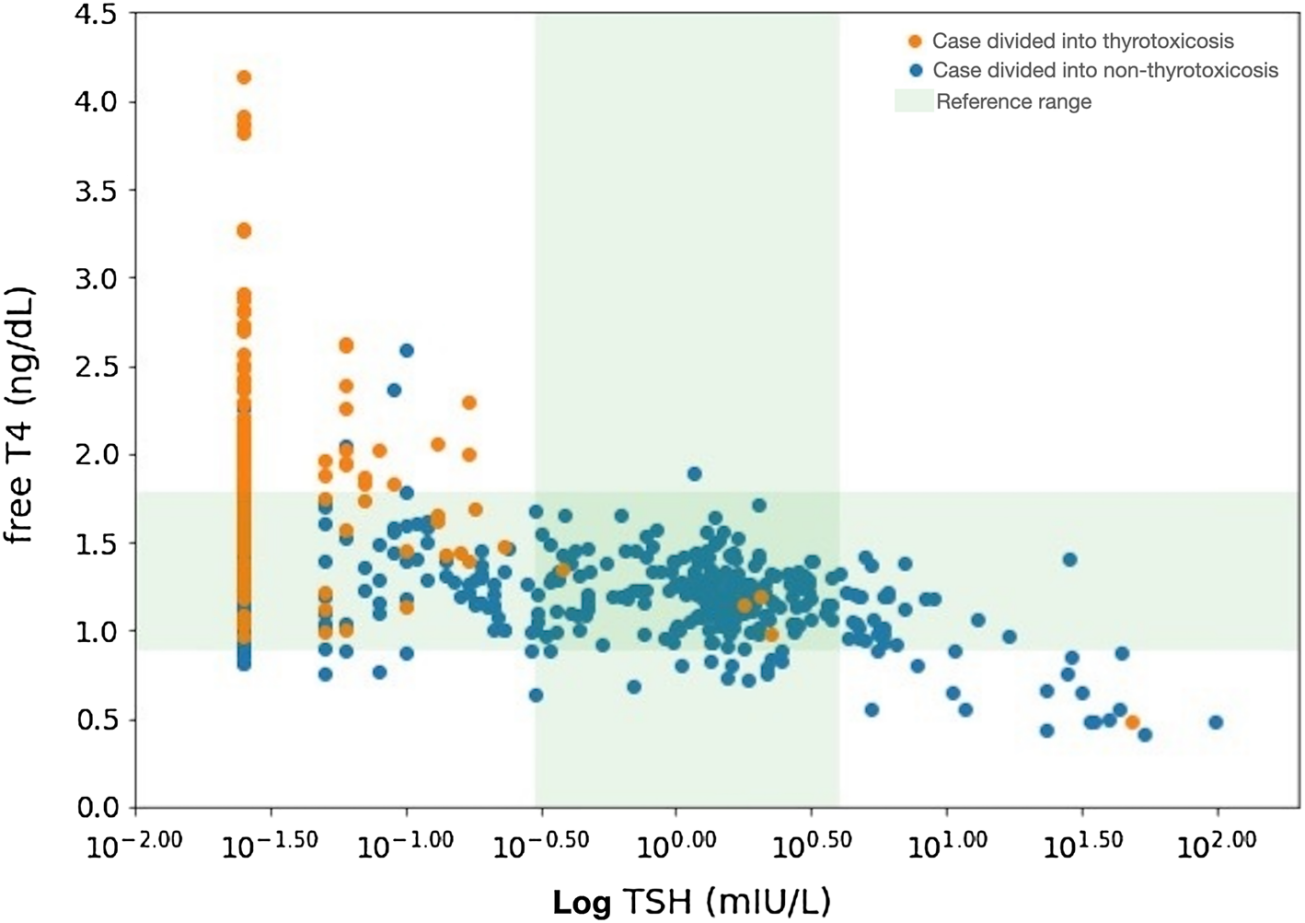
Distribution of free T4 and TSH levels of data pairs according to the decision of the ML-assisted system. *T4* thyroxine, *TSH* thyroid stimulating hormone, *ML* machine learning.

The distribution of free T4 and TSH levels of data pairs according to the decision of our ML-assisted system was demonstrated in the Fig. 1. Among 561 cases showing serum free T4 level ≤ 1.78 ng/dL, 79 cases were divided into thyrotoxicosis and 482 cases were divided into non-thyrotoxicosis. The cases divided into thyrotoxicosis showed higher free T4 and lower TSH levels compared with the cases divided into non-thyrotoxicosis (free T4, 1.48 ± 0.27 vs. 1.21 ± 0.24 ng/dL, p < 0.001; TSH, 0.025 (0.025) vs 0.755 (1.730) mIU/L, p < 0.001). The proportion of cases with TSH < 0.1 mIU/L was higher in the cases divided into thyrotoxicosis compared with cases divided into non-thyrotoxicosis (82% vs. 33%, p < 0.001).

### Collection of HR data

**Figure 2:**
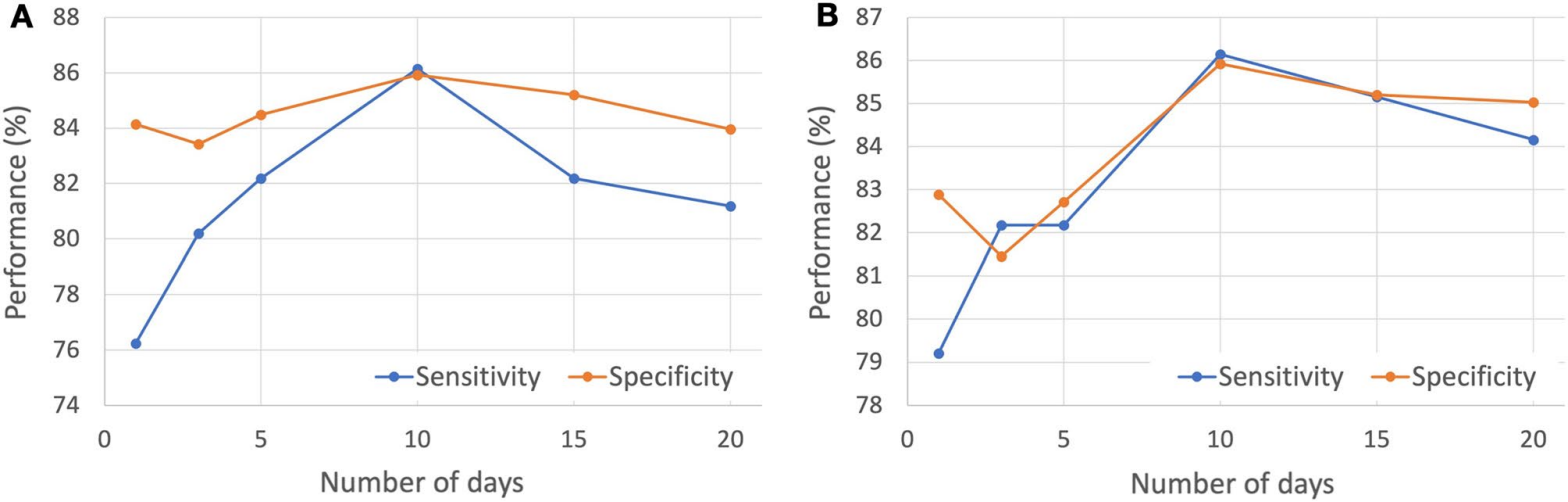
Sensitivity and Specificity for varying the number of days of collecting HR (**A**) for both training and test data and (**B**) for the test data when the duration of collecting HR data is fixed at 10 days for the training data. HR, heart rate.

In order to determine the optimal duration for collecting HR data to predict thyroid function, the diagnostic performance was measured by varying the number of days for collecting HR data. It should be noted that the use of HR data collected during N days means utilizing the HR data for N consecutive days prior to the date of referred TFT and the target date, regardless of missing values. Figure 2A displays the diagnostic performance when the duration of collecting HR data varies for both training and test data, while Fig. 2B shows the diagnostic performance when the duration of collecting HR data is fixed at 10 days for the training data and varies for the test data. Both sensitivity and specificity are highest when the HR data is collected for 10 days.

### Feature importance

**Figure 3:**
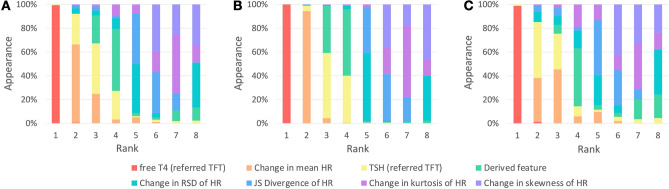
Rank for each feature in the experiment. Feature importance considering (**A**) gain and split, (**B**) gain, and (**C**) split. Gain, relative contribution of a feature in the model; Split, relative count of times a feature occurs in the model.

Figure 3 displays the feature importance of the ML-based classifiers constructed to predict the thyroid function of 662 test data. Based on the criteria of gain and split, the serum free T4 level of the referred TFT (average rank: 1.01), the change in mean HR (average rank: 2.51), and the serum TSH level of the referred TFT (average rank: 3.24) are identified as the most significant features. The average ranks of the other five features are similar.

## Discussion

In this study, a ML-assisted system was developed to predict thyrotoxicosis using HR data collected from a wearable device, which showed promising results for clinical applications. Previously, an association between serum free T4 levels and resting HR from wearable devices in patients with thyrotoxicosis was reported, where an increase of 11 beats per minute in 5-day mean resting HR was correlated with an increase in serum free T4 level of about 0.5 ng/dL and a three-fold increase in the risk of thyrotoxicosis^8^. Although a rule-based classifier using the statistical association was developed, the accuracy was not high enough for predicting thyroid function state in individuals. Therefore, ML methodology was applied, and various features of HR monitoring data were used to predict thyrotoxicosis. Although the ML-assisted system predicted thyrotoxicosis with reasonable accuracy (85.15% of sensitivity and 85.03% of specificity), it showed low PPV, indicating a high probability of false positive cases. However, most false positive cases were subclinical thyrotoxicosis, which suggests that the ML-assisted system can predict not only the current thyrotoxicosis but also the potential risk of thyrotoxicosis based on the changes of biosignals considering that subclinical thyrotoxicosis can progress to overt thyrotoxicosis^9–15^. Moreover, higher free T4 and lower TSH levels in false positive cases compared with true negative cases support that the ML-assisted system may screen more severe cases of subclinical thyrotoxicosis, which is likely to cause comorbidities^12,16–21^ and progress to overt thyrotoxicosis^13–15^.

In this study, the ML-assisted system performed best when using HR data from the 10 days preceding the date of referred TFT and target date. This result is clinically valid, as using HR data from a shorter period, such as 5 days or 1 day, may incorporate variations caused by exercise, alcohol consumption, emotional states, or other acute illnesses, potentially lowering the ML model's performance. Similarly, using HR data from a longer period than 10 days may include data distant from the date of referred TFT and target date, which could also reduce the model's performance. The essential features in our machine learning model for predicting thyroid function were the free T4 level of referred TFT and the change of mean HR. This can be reasonably predicted considering the well-known chronotropic effect of thyroid hormones on the myocardium^6^ and our previous research, which showed a correlation between resting HR collected using wearable devices and serum free T4 levels^8,22^. Therefore, our machine learning model can be considered as reflecting thyroid hormone physiology and previous clinical research results effectively.

This study has some important clinical implications. Our ML-assisted system can be integrated into a mobile app that helps patients self-manage thyroid dysfunction. In this case, the mobile app integrated with wearable devices can inform users of the risk of thyrotoxicosis and guide them to take medication diligently or encourage remote consultations or hospital visits depending on the risk level, thereby facilitating successful disease management. Additionally, our ML-assisted system can be used in remote monitoring solutions in hospitals. Although the risk of hyperthyroidism based on heart rate data from wearable devices cannot replace blood tests, our system can monitor the disease status based on the predicted risk level in patients who cannot visit the hospital or have infrequent hospital visits. The system we developed can also be used in the treatment process, such as adjusting medication. For example, it can monitor the response of anti-thyroid drug (ATD) in patients with Graves’ disease who are starting or undergoing treatment, verify the effectiveness of medication, and determine the optimal timing for hospital visits and blood tests. It can also be employed to detect disease relapse in patients with Graves’ disease who are in remission considering that approximately 50% of patients are reported to relapse within 2 years after discontinuing ATD despite following the recommended guidelines^23^. Additionally, our ML-assisted system could have implications for monitoring drug-induced subclinical thyrotoxicosis in patients with differentiated thyroid cancer (DTC) who are undergoing TSH-suppressive therapy. TSH-suppressive therapy involves maintaining a subclinical thyrotoxic state through thyroid hormone over-replacement and is recommended to prevent the growth of DTC^24^. However, excessive suppression of TSH elevates the risk of fractures and cardiovascular disease^25–27^. Therefore, a simple monitoring tool for TSH-suppressive therapy, such as a wearable device that can be used in daily life, could aid in maintaining the appropriate level of TSH suppression.

This study has some limitations which should be considered when interpreting the results. Firstly, the number of cases used in our study is relatively small compared to the number typically used for constructing ML models. This is because our study is not based on pre-existing medical databases, such as medical image readings, but on collecting biosignal data from wearable devices during unstable periods of thyroid hormone levels in patients with thyroid dysfunction. Therefore, our attempt to predict thyroid dysfunction using biosignals collected from wearable devices is unique to our research group and includes the most significant number of cases in related studies. Since we are continuously collecting data, we expect to present more advanced ML models in the future. Furthermore, since the current data is collected from a single institution, it is not immune to overfitting issues. To overcome this limitation, additional research for external validation is warranted in the future. Secondly, the data in this study predominantly collected from an age group familiar with the use of wearable devices and the associated smartphone apps. Additionally, the symptoms of thyrotoxicosis are often less apparent in the elderly population, which may limit the prediction accuracy and usability of our ML-assisted system for elderly thyrotoxicosis patients. However, in elderly patients, less evident thyrotoxic symptoms are primarily associated with mood-related symptoms, and even in elderly patients diagnosed with apathetic hyperthyroidism, the effects of thyroid hormones on the heart are not different from those in the general population with typical hyperthyroidism, as increased heart rate is observed^28–30^. Furthermore, according to a study conducted in Austria that measured resting heart rate in patients undergoing coronary angiography, it was observed that the positive correlation between resting heart rate and fT4 levels remains consistent even in relatively older patients with an average age of 62.8 years, indicating the presence of this relationship in an older age group^31^. Therefore, it is expected that the performance of our ML-assisted system will be maintained in elderly patients as well.

In conclusion, a ML-assisted system has made reasonably accurate predictions of thyrotoxicosis in patients with thyroid dysfunction. The accuracy of the system could be further improved by obtaining more data. This predictive system can be used for monitoring patients' thyroid function status without requiring blood tests, either as a smartphone-based self-monitoring tool or as a remote monitoring solution in hospitals.

## Subjects and methods

### Study design and participants

This is a retrospective cohort study that combines data from three different studies: a proof study that demonstrated the clinical feasibility of monitoring resting HR using a wearable device in patients with thyrotoxicosis^8^, an extension study that collected additional data on patients with thyroid dysfunction, and an app-feasibility study that evaluated the clinical feasibility of a disease-managing mobile application for thyroid dysfunction. Participants in the proof study were recruited from the outpatient clinic of the endocrinology department at Seoul National University Bundang Hospital (SNUBH) between November 2016 and June 2017. Patients between the ages of 15 and 60 who had been newly diagnosed with or had a recurrence of thyrotoxicosis were eligible to participate. The extension study, which aimed to collect more data, recruited patients between the ages of 18 and 60 who had been newly diagnosed with thyroid dysfunction, including thyrotoxicosis or hypothyroidism, or were being treated for thyroid dysfunction. This study began recruiting participants at SNUBH in January 2021, and recruitment is ongoing. The feasibility study to evaluate the disease-managing mobile application recruited patients aged 18 years and older who had been newly diagnosed with Graves' disease at SNUBH from March 2022, and recruitment is also ongoing. The data from all three studies were combined to develop a machine learning (ML)-assisted system to predict thyrotoxicosis. All participants included in these studies needed to own a smartphone and to be able to use a wearable device and its mobile application. TFT were performed more than 3 times at more than 4-week intervals in the participants and their activity, sleep, and HR data were continuously monitored with a wearable device during the study period. In these studies, participants utilized a mobile application for self-management of thyroid dysfunction, Glandy™ (THYROSCOPE INC., Ulsan, Republic of Korea), to manage the data collected from wearable devices. Prior to using the app, participants agreed to the terms and conditions and the privacy policy and provided their clinical information, including data collected from their wearable devices and results from thyroid function tests. Personal identifying information was not provided, and the analysis of data was conducted using only the information that participants agreed to provide. The studies included in this retrospective study were approved by the SNUBH Institutional Review Board (IRB number, B-1609-363-004, B-2012-654-303, and B-2201-735-304) and registered on Clinicaltrials.gov (trial registration number, NCT03009357, NCT04806269, and NCT05828732). All methods were carried out in accordance with relevant guidelines and regulations and informed consent was obtained from all subjects.

### Wearable devices and applications

In the proof study^8^, we used the Fitbit Charge HR™ or Fitbit charge 2™ (Fitbit, San Francisco, CA) and the Fitbit application for iOS™ (Apple, Cupertino, CA) or Android™ (Google, Mountain View, CA). The firmware versions of these devices were 18.128 for Fitbit charge HR™ and 22.53.4 for Fitbit charge 2™ at the end of the study, and the latest version was maintained continuously over the study period. In the extension study and the app-feasibility study, we used Fitbit inspire™ or Fitbit inspire 2™ (Fitbit) and the Fitbit application for iOS™ (Apple) or Android™ (Google). The firmware versions of these devices were updated to the latest version during the study period (1.88.11 for Fitbit inspire™ and 1.124.28 for Fitbit inspire 2™). All models share a common sensor and data processing algorithm for both activity tracking and HR measurement. Activity and HR data are collected by the 3-axis accelerometer and photoplethysmography sensor, respectively. Fitbit also provide sleep data, including total time asleep and total number and time of awakening using their own algorithm to detect sleep from activity and HR data. To collect interday summarized data of activity, HR, and sleep and intraday detailed data of activity and HR, we used a mobile application for self-management of thyroid dysfunction, Glandy™ (Thyroscope inc., Ulsan, Republic of Korea) for iOS™ (Apple) or Android™ (Google). This mobile application uses the application programming interface provided by Fitbit and makes it possible to collect the data mentioned above after Fitbit user’s authorization.

### Biochemical measurements

Serum concentrations of free T4 and thyroid-stimulating hormone (TSH) were measured using immunoassays (free T4: DiaSorin S.p.A.; TSH: CIS Bio International). The free T4 assay had an analytic sensitivity of 0.05 ng/dL, and TSH had an analytical sensitivity of 0.04 mIU/L and functional sensitivity of 0.07 mIU/L. The reference ranges for free T4 and TSH were 0.89–1.78 ng/dL and 0.3–4.0 mIU/L, respectively. Thyroid function status was defined based on the results of the TFT. Overt thyrotoxicosis was defined as higher free T4 level than reference ranges; subclinical thyrotoxicosis as normal free T4 and lower TSH levels; euthyroid as normal free T4 and TSH; subclinical hypothyroidism as normal free T4 and higher TSH levels; overt hypothyroidism as lower free T4 level.

### Data preparation

We utilized a dataset consisting of “HR-TFT pairs”, paired TFT results and HR data measured using a wearable device over a period of 10 days. Specifically, we paired the result of the TFT, which is carried out in the daytime, with the sleeping heart rates measured during the 10 days prior to the test date. For each day, a series of sleeping heart rates for 1 sleep session belongs to the date when the sleep ends. We extracted these HR-TFT pairs from a total of 175 patients and a total of 662 HR-TFT pairs were collected. Among these, the number of euthyroid, subclinical thyrotoxicosis, and overt thyrotoxicosis based on TFT results were 229, 263, and 101 pairs, respectively (Table 3). Because we included the subjects with thyrotoxicosis and hypothyroidism in the extension study, 36 data pairs of overt hypothyroidism and 33 data pairs of subclinical hypothyroidism were included in the dataset (Table 3). The HR data which we used was detailed HR data during sleep based on each user’s sleep time data provided by Fitbit.

**Table 3:** Participants and data characteristics.

Age (year)	38.9 ± 9.2
Sex (male/female)	28/147
Thyroid function state of data	
Overt thyrotoxicosis (n = 101)	
Free T4 (ng/dL)	2.51 ± 1.30
TSH (mIU/L)	0.049 (0.000)
Subclinical thyrotoxicosis (n = 263)	
Free T4 (ng/dL)	1.37 ± 0.24
TSH (mIU/L)	0.055 (0.025)
Euthyroid (n = 229)	
Free T4 (ng/dL)	1.22 ± 0.16
TSH (mIU/L)	1.488 (0.900)
Subclinical hypothyroidism (n = 33)	
Free T4 (ng/dL)	1.11 ± 0.15
TSH (mIU/L)	6.995 (1.730)
Overt hypothyroidism (n = 36)	
Free T4 (ng/dL)	0.70 ± 0.14
TSH (mIU/L)	16.236 (28.905)

Data are expressed as mean ± SD or median (interquartile range). *T4* thyroxine, *TSH* thyrotropin.

### Development of the ML-assisted system

**Figure 4:**
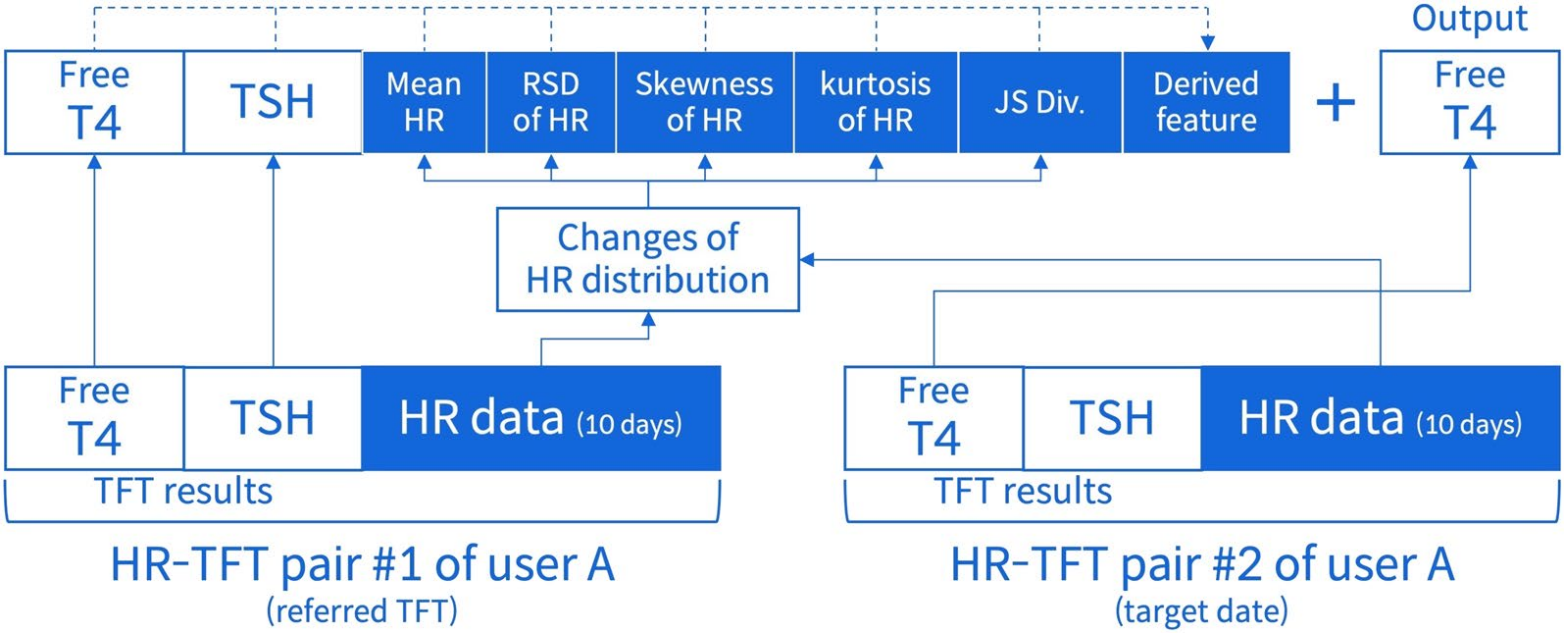
Design of a ML-assisted system to predict the occurrence of thyrotoxicosis using HR data collected from wearable devices. *ML* machine learning, *HR* heart rate, *T4* thyroxine, *TSH* thyroid stimulating hormone, *RSD* relative standard deviation, *JS Div* Jensen–Shannon divergence, *TFT* thyroid function test.

The proposed system is designed to classify whether a user has thyrotoxicosis by using the relationship between the change of serum free T4 level and the change of HR data, where the relationship is represented by the data combining each two HR-TFT pairs (one for the date of the referred TFT and another for the target date) of a user. The input of the system consists of the results of the referred TFT (i.e., serum free T4 level and serum TSH level) and the change of HR data between the two different time points, while the binary output of the system indicates whether the user has thyrotoxicosis. The data with free T4 level > 1.78 ng/dL at the target date is labeled as positive. As shown in Fig. 4, in the input, the change of HR data is expressed with five features (changes in mean HR, changes in the relative standard deviation of HR, changes in the skewness of HR, changes in the kurtosis of HR, Jensen-Shannon divergence) to utilize the difference between two distributions of HR data. Additionally, we newly derived one feature from existing ones (e.g., product/quotient of two different features) to enhance the prediction. Among candidates, we selected the quotient of changes in mean HR divided by TSH level of referred TFT.

Light gradient boosting machine, tree based gradient boosting algorithm, is used to establish a classifier, and each input feature is transformed by using quantile transformer. The training data are augmented by creating HR-TFT pairs consisting of free T4 levels and TSH levels calculated by linear interpolation of those of two adjacent TFTs, and actual HR data of corresponding dates. During our training process, we combined data from two time points of a single patient to create one case for training. As a result, with the initial 662 HR-TFT pairs from 175 individuals, we had a total of 2182 cases for training. When including the additional data generated through interpolation, we were able to use a total of 2,711 HR-TFT pairs, resulting in a pool of 31,138 cases available for training.

### Statistical analysis

Values with a normal distribution are expressed as mean ± SD, and values with a non-normal distribution are expressed as median (interquartile range). We used Student t test or the Mann Whitney U test for continuous variables. The sensitivity, specificity, positive predictive value (PPV), and negative predictive value (NPV) of the ML-assisted system for detecting thyrotoxicosis were computed based on the definition of overt thyrotoxicosis. Diagnostic values ​were analyzed using leave one-out cross validation (LOOCV), but the LOOCV was modified so that the training data did not include the test data. For example, if we have two participants A and B, and these participants have 3 HR-TFT pairs at three different time points (T1, T2, and T3). Our ML-assisted system predicts thyrotoxicosis in participant A at T2 using HR data at T2 and the HR-TFT pair at T1 (Ta1 → Ta2) or T3 (Ta3 → Ta2). When we test Ta1 → Ta2, learning data are as follows; Ta1 → Ta3, Ta3 → Ta1, Tb1 → Tb2, Tb2 → Tb1, Tb1 → Tb3, Tb3 → Tb1, Tb2 → Ta3, and Tb3 → Tb2). We did not include Ta3 → Ta2 in the training data to prevent the inclusion of the test data in the training data. Our ML-assisted system predicts thyrotoxicosis at T2 in participant A with the average value of probability derived from Ta1 → Ta2 and Ta3 → Ta2. A two-tailed *p* < 0.05 was considered statistically significant. All statistical analyses and data preparation were performed using IBM SPSS Statistics (version 28.0; IBM Corporation, Armonk, NY, USA).

## Data Availability

The raw data including medical records are not available upon request due to ethical and legal restrictions imposed by the SNUBH IRB. The original data are derived from the institutions’ electronic health records and contain patients’ protected health information. Deidentified data are available from the SNUBH for researchers who meet the criteria for access to confidential data and have a data usage agreement with the hospital. Data requests for this study should be made by contacting the corresponding author, Jae H.M.

## Abbreviations

ATD: Anti-thyroid drug
HR: Heart rate
LOOCV: Leave one-out cross validation
ML: Machine learning
NPV: Negative predictive value
PPV: Positive predictive value
SNUBH: Seoul National University Bundang Hospital
T3: Triiodothyronine
T4: Thyroxine
TFT: Thyroid function test
TSH: Thyroid stimulating hormone
